# Adding Celecoxib With or Without Zoledronic Acid for Hormone-Naïve Prostate Cancer: Long-Term Survival Results From an Adaptive, Multiarm, Multistage, Platform, Randomized Controlled Trial

**DOI:** 10.1200/JCO.2016.69.0677

**Published:** 2017-03-13

**Authors:** Malcolm D. Mason, Noel W. Clarke, Nicholas D. James, David P. Dearnaley, Melissa R. Spears, Alastair W.S. Ritchie, Gerhardt Attard, William Cross, Rob J. Jones, Christopher C. Parker, J. Martin Russell, George N. Thalmann, Francesca Schiavone, Estelle Cassoly, David Matheson, Robin Millman, Cyrill A. Rentsch, Jim Barber, Clare Gilson, Azman Ibrahim, John Logue, Anna Lydon, Ashok D. Nikapota, Joe M. O’Sullivan, Emilio Porfiri, Andrew Protheroe, Narayanan Nair Srihari, David Tsang, John Wagstaff, Jan Wallace, Catherine Walmsley, Mahesh K.B. Parmar, Matthew R. Sydes, for the STAMPEDE Investigators

**Affiliations:** Malcolm D. Mason, Cardiff University School of Medicine, Velindre Hospital; Jim Barber, Velindre Cancer Centre, Cardiff; Noel W. Clarke, The Christie and Salford Royal NHS Foundation Trusts; John Logue, Christie Hospital, Manchester; Nicholas D. James, Institute of Cancer and Genomic Sciences; Emilio Porfiri, The Medical School, University of Birmingham; Nicholas D. James, Queen Elizabeth Hospital; Emilio Porfiri, University Hospitals Birmingham NHS Foundation Trust, Birmingham; David P. Dearnaley, Gerhardt Attard, and Christopher C. Parker, The Institute of Cancer Research and Royal Marsden NHS Foundation Trust; Melissa R. Spears, Alastair W.S. Ritchie, Francesca Schiavone, David Matheson, Robin Millman, Clare Gilson, Mahesh K.B. Parmar, and Matthew R. Sydes, MRC Clinical Trials Unit at UCL, London; William Cross, Leeds Teaching Hospitals NHS Trust, Leeds; Rob J. Jones and J. Martin Russell, University of Glasgow; Rob J. Jones and Jan Wallace, Beatson West of Scotland Cancer Centre, Glasgow; Azman Ibrahim, The Clatterbridge Cancer Centre NHS Foundation Trust, Bebington, Wirral; Anna Lydon, Torbay District Hospital, Torquay; Ashok D. Nikapota, Sussex Cancer Centre, Brighton; Ashok D. Nikapota, Worthing Hospital, Worthing; Joe M. O’Sullivan, Centre for Cancer Research and Cell Biology, Queen's University, Belfast; Andrew Protheroe, Churchill Hospital, Oxford; Narayanan Nair Srihari, Shrewsbury and Telford Hospitals NHS Trust, Shrewsbury; David Tsang, Southend Hospital, Southend-on-Sea; David Tsang, Basildon Hospital, Basildon; John Wagstaff, The South West Wales Cancer Institute; John Wagstaff, Swansea University College of Medicine, Swansea; Catherine Walmsley, Royal Preston Hospital, Preston, United Kingdom; George N. Thalmann, University Hospital; Estelle Cassoly, SAKK Coordinating Center, Berne; and Cyrill A. Rentsch, University Hospital Basel, Basel, Switzerland.

## Abstract

**Purpose:**

Systemic Therapy for Advanced or Metastatic Prostate Cancer: Evaluation of Drug Efficacy is a randomized controlled trial using a multiarm, multistage, platform design. It recruits men with high-risk, locally advanced or metastatic prostate cancer who were initiating long-term hormone therapy. We report survival data for two celecoxib (Cel)-containing comparisons, which stopped accrual early at interim analysis on the basis of failure-free survival.

**Patients and Methods:**

Standard of care (SOC) was hormone therapy continuously (metastatic) or for ≥ 2 years (nonmetastatic); prostate (± pelvic node) radiotherapy was encouraged for men without metastases. Cel 400 mg was administered twice a day for 1 year. Zoledronic acid (ZA) 4 mg was administered for six 3-weekly cycles, then 4-weekly for 2 years. Stratified random assignment allocated patients 2:1:1 to SOC (control), SOC + Cel, or SOC + ZA + Cel. The primary outcome measure was all-cause mortality. Results were analyzed with Cox proportional hazards and flexible parametric models adjusted for stratification factors.

**Results:**

A total of 1,245 men were randomly assigned (Oct 2005 to April 2011). Groups were balanced: median age, 65 years; 61% metastatic, 14% N+/X M0, 25% N0M0; 94% newly diagnosed; median prostate-specific antigen, 66 ng/mL. Median follow-up was 69 months. Grade 3 to 5 adverse events were seen in 36% SOC-only, 33% SOC + Cel, and 32% SOC + ZA + Cel patients. There were 303 control arm deaths (83% prostate cancer), and median survival was 66 months. Compared with SOC, the adjusted hazard ratio was 0.98 (95% CI, 0.80 to 1.20; *P* = .847; median survival, 70 months) for SOC + Cel and 0.86 (95% CI, 0.70 to 1.05; *P* =.130; median survival, 76 months) for SOC + ZA + Cel. Preplanned subgroup analyses in men with metastatic disease showed a hazard ratio of 0.78 (95% CI, 0.62 to 0.98; *P* = .033) for SOC + ZA + Cel.

**Conclusion:**

These data show no overall evidence of improved survival with Cel. Preplanned subgroup analyses provide hypotheses for future studies.

## INTRODUCTION

Systemic Therapy for Advanced or Metastatic Prostate Cancer: Evaluation of Drug Efficacy (STAMPEDE) is a multiarm, multistage (MAMS), platform, randomized controlled trial. Its novel design^1,2^ allowed simultaneous assessment of adding various therapies to standard of care (SOC; androgen deprivation). The trial recruited patients commencing long-term hormone therapy (HT) for high-risk, locally advanced, or metastatic prostate cancer (CaP), either newly diagnosed or after failure of previous local therapy. Results from STAMPEDE’s docetaxel comparison showed major improvements in survival.^3^ A companion meta-analysis^4^ combining data from other major international trials^5,6^ confirmed the usefulness of that combination, changing world-wide practice.^7,8^ Here, we report outcomes after SOC plus either celecoxib (Cel) or Cel and zoledronic acid (ZA; Data Supplement).

Cox-2 inhibition is associated with inhibition of carcinogenesis,^9-12^ and case-control studies have shown a reduced risk of CaP.^13-15^ ZA has known anti-CaP effects, demonstrated both clinically in later-stage disease^16^ and in vitro.^17^ The first-generation bisphosphonate clodronate improved survival when used concurrently with long-term HT for metastatic CaP.^18^ The anticipated mechanisms of action of Cox-2 inhibitors such as Cel and bisphosphonates such as ZA were considered complementary, allowing targeting of both bone progression and the underlying molecular changes that lead to progression.

The MAMS design uses increasingly stringent hurdles at interim analyses to determine whether recruitment to a comparison should continue through to fully powered survival analysis. Interim analysis was performed on failure-free survival (FFS), primarily driven by rising prostate-specific antigen (PSA).

In 2011, at the second, preplanned activity analysis, the Independent Data Monitoring Committee reviewed data, including those on toxicity and FFS. The observed safety of Cox-2 inhibition ± ZA was not questioned; closure to recruitment to both Cel-containing arms was recommended because of insufficient activity on FFS, guided by a protocol-defined activity target of a hazard ratio (HR) of 0.92. The Trial Steering Committee agreed that Cel should be stopped in both arms. The ZA was continued because the ZA comparison was continuing. On the committee’s recommendation, comparative FFS data for the Cel-only arm were published; follow-up continued as planned.^19^

Release of survival data was intended to follow publication of survival data for the docetaxel, ZA, and docetaxel + ZA comparisons, which started recruitment simultaneously and passed through all intermediate analyses.^3^ For the purposes of understanding, we also include some information on contemporaneously randomly assigned patients allocated to SOC + ZA; this is updated information on a subset of patients reported previously.^3^ We also contextualize our findings on Cel + ZA with summary information from the docetaxel arms in STAMPEDE, because that treatment is now an increasingly used SOC.

## PATIENTS AND METHODS

The trial, detailed previously,^2,3,19-20^ was run according to Good Clinical Practice guidelines and the Declaration of Helsinki, with relevant regulatory and ethics approvals.

### Study Design and Participants

A MAMS platform approach was used.^1,21^ Eligible patients had CaP that was newly diagnosed and either metastatic, node positive, or high-risk locally advanced (with ≥ 2 of T3/4, Gleason 8 to 10, and PSA ≥ 40 ng/mL). Eligibility criteria also included that the patients had been treated previously with radical surgery or radiotherapy (RT) now relapsing with high-risk features. All were initiating long-term HT within 12 weeks before random assignment. Patients were required to be fit for chemotherapy with no history of severe cardiovascular disease. All gave written informed consent.

### Random Assignment and Masking

A computerized algorithm implemented minimization-based random assignment (random element, 20%), stratifying for hospital, age, presence of metastases, planned RT use, nodal involvement, WHO performance status, planned HT type, and regular aspirin and/or nonsteroidal anti-inflammatory drug (NSAID) use. Allocation was 2:1:1 for open-label SOC-only, SOC + Cel, and SOC + ZA + Cel.

### Treatment

HT was lifelong for patients with metastatic disease and ≥ 2years for patients with nonmetastatic disease. HT was with gonadotropin-releasing hormone agonists or antagonists or orchidectomy; patients with nonmetastatic disease could receive oral anti-androgens alone; patients with metastatic disease could undergo orchidectomy. RT was encouraged for patients with nonmetastatic disease 6 to 9 months after random assignment.^22^

Cel was given orally 400 mg twice a day for 1 year after regulatory authority advice after withdrawal of another Cox-2 inhibitor, rofecoxib. ZA was planned for ≤ 2 years, given as outpatient infusions at 4 mg/15 min on approximately 66 occasions, starting once every 3 weeks for six cycles then once every 4 weeks. The protocol described modifications for adverse events (AEs). Either treatment was to stop for intolerable AEs or an FFS event.

### Follow-Up

Follow-up, including PSA tests, was every 6 weeks for 6 months, then every 12 weeks to 2 years, every 6 months to 5 years, then annually. Additional tests were at the investigators’ discretion. Common Toxicity Criteria version 3.0^1^ was used to grade AEs; serious AEs were reported promptly by sites.

### Primary and Secondary Outcomes

The primary outcome measure, survival, was time from random assignment to death from any cause. The intermediate primary outcome measure, FFS, was time from random assignment to first evidence of either biochemical failure; local, lymph node, or distant metastatic progression; or death as a result of CaP. Biochemical failure was determined as a rise in PSA ≥ 4 ng/mL and 50% above the lowest reported PSA within 24 weeks after random assignment, or failure to decrease by 50% from the starting PSA during this time. When possible, the cause of death was determined by blinded central review. Death as a result of CaP was recorded when classified by the reviewer as definitely or probably CaP; the site investigator's determination was used for 11 of 584 deaths (2%), with insufficient data available for central review; deaths without reported cause were classified as non-CaP.

### Statistical Design and Analysis

We assumed a median FFS of 2 years and a survival of 4 to 5 years for control subjects and targeted a 25% relative improvement (HR, 0.75) in FFS and overall survival (OS) for each pairwise comparison of research arm to control arm. The Stata program nstage (Stata, College Station, TX) allowed the MAMS design with three intermediate activity analyses on the basis of FFS and an efficacy analysis on the basis of survival. The latter had 90% power and a 2.5% one-sided α, requiring approximately 400 control arm deaths; the former each had 95% power and increasingly stringent one-sided αs of 50%, 25%, and 10%, requiring approximately 114, 216, and 334 FFS events, respectively, and expressed as lack-of-benefit stopping guidelines.^19^ Only patients randomly assigned contemporaneously were compared head to head for each pairwise comparison. Patients were included in the efficacy analyses according to allocated treatment on an intention-to-treat (ITT) basis, unless stated.

Accumulating data were reviewed by the Independent Data Monitoring Committee. Patients allocated to the Cel arm who still met the eligibility criteria when accrual stopped prematurely were offered complete withdrawal and re-random assignment to an ongoing arm (this is not one of the primary comparisons reported here); five accepted and contribute here only a short period of data from random assignment to withdrawal from their original allocation. Standard survival analysis methods were used for analyses of time-to-event data in STATA version 14. Patients without the relevant event were censored when last reported event free. Cox proportional hazards regression models, adjusted for stratification factors (with the exception of hospital and planned HT), were used to estimate most relative treatment effects, with HR < 1.00 favoring the research arm. Flexible parametric models^23^ with five degrees of freedom for the baseline hazard function and five degrees of freedom for the time-dependent effect and adjusted for stratification factors were used to estimate medians, 5-year event rates, and restricted mean survival time. Restricted mean survival time took primacy if there was evidence of nonproportional hazards. The likelihood ratio test was used to test for the presence of treatment-subgroup interactions. Fine and Gray regression models were used for competing risk analysis of CaP-specific survival. All confidence intervals are at 95%. Prespecified analyses examined consistency of treatment effect by stratification factors, categorized Gleason score (≤ 7, ≥ 8, or unknown), timing of random assignment (newly diagnosed or recurrent after previous local therapy), and, as a continuous variable, pre-HT PSA. A *P* value < .10 was taken to be indicative of possible heterogeneity. A preplanned subgroup analysis of M1 patients at random assignment was included in the statistical analysis plan and the complementary M0 group. Sensitivity analyses dividing patients by whether they could have received the maximal duration of Cel (earlier), or not (later), were considered (Data Supplement).

Median follow-up was estimated by reverse censoring on death, in which survival is treated as the event and death as censoring.

Preplanned exploratory factorial analyses of ZA and Cel, with and without an interaction term, drew in data from those patients randomly assigned contemporaneously to SOC + ZA.

The safety population for analysis of AEs grouped patients according to treatment started, with sensitivity analysis on an ITT basis. Data on first reported symptomatic skeletal event (SSE) and osteonecrosis of the jaw are also presented.

## RESULTS

### Accrual and Patient Characteristics

**Fig 1.**
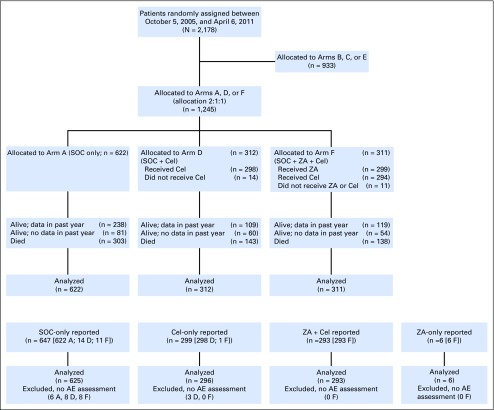
CONSORT flow diagram depicting the flow of patients who joined the STAMPEDE trial while these specific comparisons were open to recruitment. Further context is given in the Data Supplement. A, standard of care (SOC); AE, adverse event; Cel, celecoxib; D, SOC + Cel; F, ZA + SOC + Cel; ZA, zoledronic acid.

Between October 5, 2005, and April 6, 2011, 1,245 men were randomly assigned 2:1:1 from 80 sites in the United Kingdom and two sites in Switzerland: 622 to SOC-only, 312 to SOC + Cel, and 311 to SOC + ZA + Cel. Data were frozen on December 15, 2015. Figure 1 presents the CONSORT flow diagram and Table 1, baseline characteristics; the Data Supplement contextualizes these arms overall. Median follow-up was 69 months. Median age was 65 years and median PSA was 66 ng/mL, and 65% had a Gleason sum score of 8 to 10. One thousand one hundred sixty-six patients (94%) were newly diagnosed, and 717 (61%) were metastatic at entry; 38 of 79 (48%) who were recurrent after local treatment had metastases.

**Table 1.**
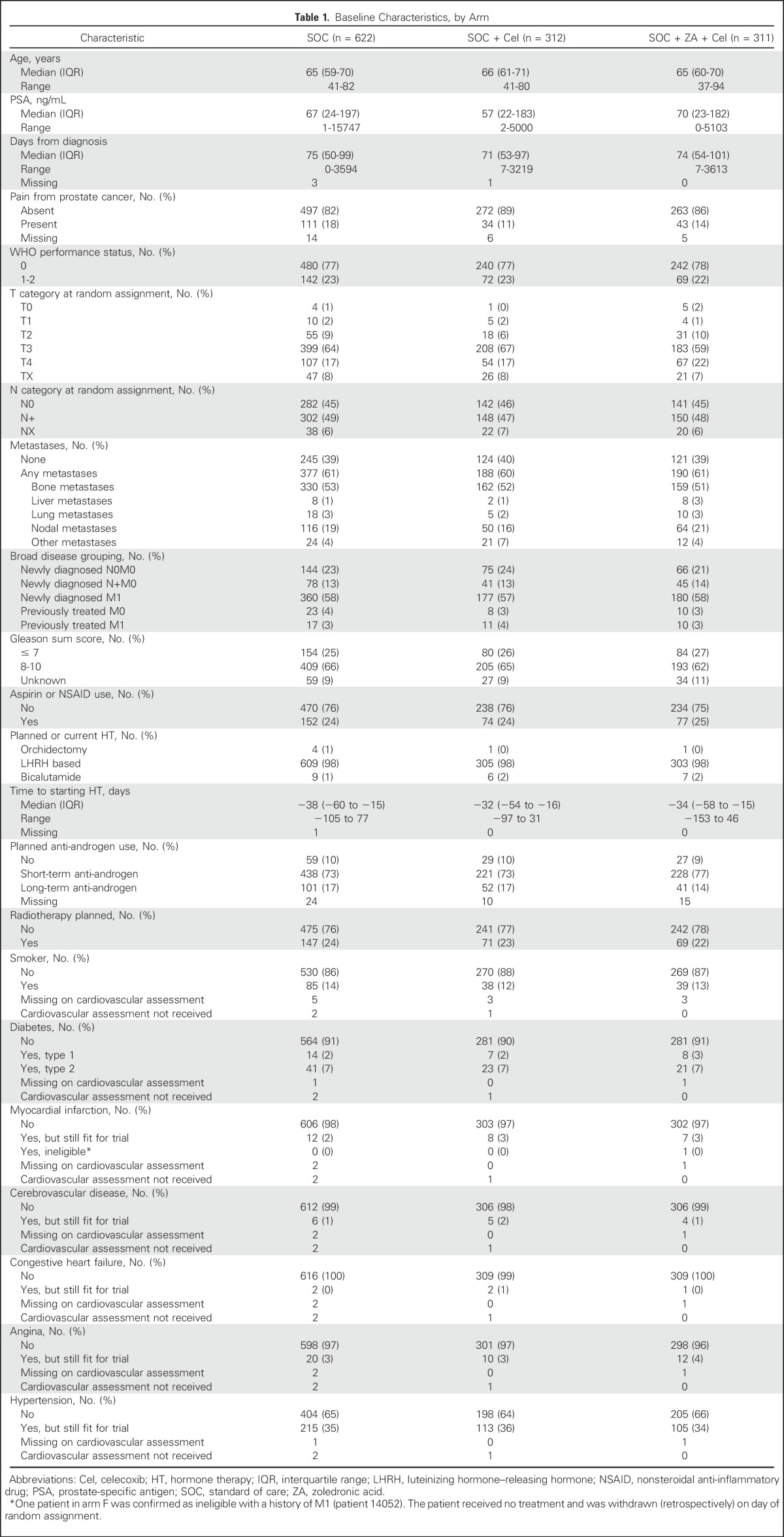
Baseline Characteristics, by Arm

### Treatment

For SOC + Cel and SOC + ZA + Cel, respectively, median time to starting Cel was 0.9 and 1.7 weeks after random assignment, and 6.5 and 7.1 weeks after starting HT; 14 and 17 patients, respectively, did not report starting Cel. Median duration of Cel was 8.3 months for SOC + Cel and 8.0 months for SOC + ZA + Cel (Data Supplement). The most common reason for stopping was treatment completion: 120 of 298 (40%) and 116 of 293 (40%), respectively (Data Supplement).

Median ZA starting time was 1.7 weeks after random assignment, and 7.1 weeks from starting HT. ZA was not reported as having started in 12 patients. Median ZA duration was 15 months (Data Supplement). Progression was the most common reason (130 of 297) for stopping treatment early; 100 of 297 (34%) completed the planned 2 years (Data Supplement).

SOC RT was reported in 158 of 622 SOC-only (25%), 70 of 312 SOC + Cel (22%), and 56 of 311 SOC + ZA + Cel (18%). In patients with nonmetastatic disease, 140 of 245 (57%), 54 of 124 (44%), and 51 of 121 (42%), respectively, reported primary site RT (Data Supplement).

### Survival

**Fig 2.**
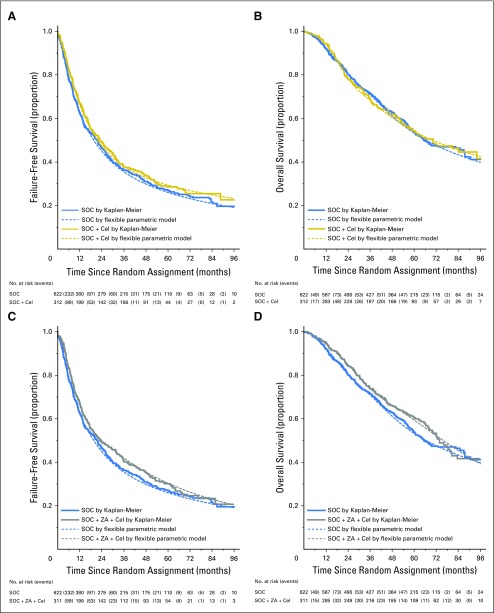
Failure-free and overall survival, by research comparison. Kaplan-Meier plots showing time to event for the definitive primary outcome measure (overall survival) and intermediate primary outcome measure (failure-free survival). (A) Failure-free survival in the celecoxib comparison. (B) Overall survival in the celecoxib comparison. (C) Failure-free survival in the ZA + celecoxib comparison. (D) Overall survival in the ZA + Cel comparison. Cel, celecoxib; SOC, standard of care; ZA, zoledronic acid.

There were 303 deaths (251 deaths related to CaP; 83%) in the SOC-only group; median survival was 66 months; and 5-year survival was 53%. There was no evidence of a survival advantage for SOC + Cel (HR, 0.98; 95% CI, 0.80 to 1.20; *P* = .847: 143 deaths [117 deaths related to CaP; 82%]); median survival was 70 months; and 5-year survival was 54%. Nor was there evidence of a survival advantage for SOC + ZA + Cel (HR, 0.86; 95% CI, 0.70 to 1.05; *P* = .130: 138 deaths [103 deaths related to CaP; 75%]); median survival was 76 months; and 5-year survival was 59% (Figs 2B and 2D). There was no evidence of nonproportional hazards data.

**Fig 3.**
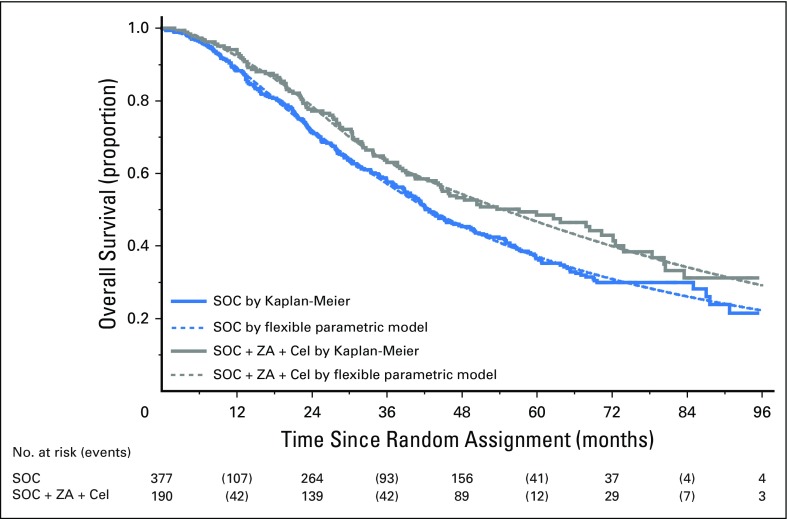
Overall survival for SOC + Cel + ZA versus SOC in patients with metastatic disease. Kaplan-Meier plot showing overall survival for the ZA + Cel comparison in patients who presented with metastatic disease at random assignment. Cel, celecoxib; SOC, standard of care; ZA, zoledronic acid.

**Fig 4.**
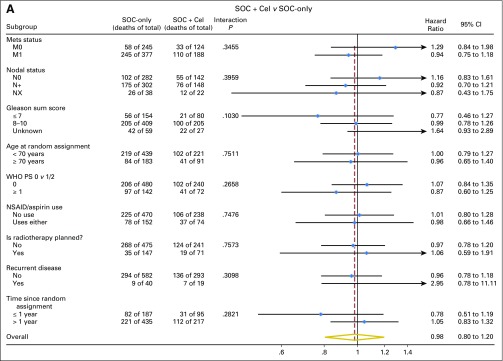

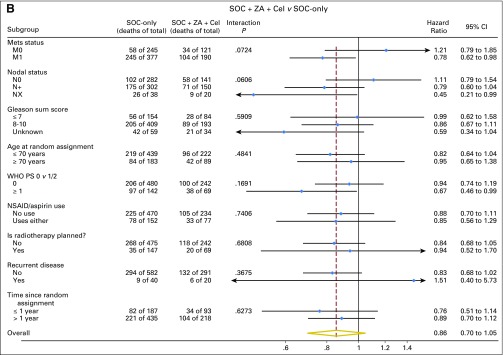
Forest plots of treatment effect on survival within subgroups, by research comparison, showing assessment of consistency of the treatment effect on overall survival in preplanned subgroups for (A) the Cel comparison and (B) the ZA + Cel comparison. The number of deaths and the number of patients are shown by arm for each treatment level, together with an adjusted hazard ratio and a test for heterogeneity of the treatment effect. Cel, celecoxib; Mets, metastases; NSAID, nonsteroidal anti-inflammatory drug; PS, performance status; RT, radiotherapy; SOC, standard of care; ZA, zoledronic acid.

Preplanned subgroup analyses in 755 M1 patients included 355 and 349 deaths for the two comparisons. This included 245 deaths in SOC-only; median survival was 43 months; and 5-year survival was 37%. There were 110 deaths in the SOC + Cel group (HR, 0.94; 95% CI, 0.75 to 1.18; *P* = .602); median survival was 43 months; and 5-year survival was 40%. There were 104 deaths in the SOC + ZA + Cel group (HR, 0.78; 95% CI, 0.62 to 0.98; *P* = .033); median survival was 55 months; and 5-year was survival 47% (Fig 3). Similar comparisons in M0 patients are relatively immature, with < 100 deaths per comparison. However, we found some indication of possible heterogeneity of treatment effect by metastatic status at random assignment for SOC + ZA + Cel (*P* = .072; Fig 4). Apart from nodal status (*P* = .061), there was no other evidence of heterogeneity of treatment effect for either comparison (Fig 4).

Exploratory analysis of the main effects of Cel and ZA individually in a single factorial model without a treatment-interaction term did not associate either drug with a survival advantage (Cel HR, 0.97; 95% CI, 0.83 to 1.13; *P* = .670; and ZA HR, 0.89; 95% CI, 0.77 to 1.04; *P* = .150). The factorial model, including a treatment-interaction term, found no evidence of treatment interaction (*P* = .788).

In patients with metastatic disease, the single factorial model without a treatment-interaction term showed no advantage to Cel (HR, 0.92; 95% CI, 0.77 to 1.09; *P* = .341) or ZA (HR, 0.86; 95% CI, 0.72 to 1.02; *P* = .083). A further exploratory factorial model, including a treatment-interaction term, found no evidence of treatment interaction (*P* = .748).

### FFS

Figures 2A and 2C present an FFS plot for each comparison. PSA failure was the most common for each arm (Data Supplement). There were 457 FFS events for SOC-only; median FFS was 20 months; and 5-year FFS was 26%. SOC + Cel had 213 events with no evidence of improved FFS (HR, 0.87; 95% CI, 0.74 to 1.03; *P* = .102); median FFS was 22 months; and 5-year FFS was 29%. SOC + ZA + Cel had 213 events and some evidence of a difference compared with SOC (HR, 0.84; 95% CI, 0.72 to 0.99; *P* = .043); median FFS was 24 months; and 5-year FFS was 30%. There was no evidence of nonproportional hazards.

There was evidence of heterogeneity of treatment effect by predefined subgroups including performance status and baseline NSAID and/or aspirin use for both comparisons, in addition to recurrent disease for SOC + Cel and Gleason score and age at random assignment for SOC + ZA + Cel (Data Supplement). In the subgroup analyses by baseline metastatic status, the estimates for FFS in SOC + ZA + Cel were HR, 0.77 (95% CI, 0.63 to 0.93) in metastatic disease and HR, 1.02 (95% CI, 0.75 to 1.39) in nonmetastatic disease, but there was no evidence of heterogeneity (*P* = .119).

Factorial analyses without an interaction term in the 643 earlier patients suggested effects on FFS in patients with metastatic disease from both Cel (HR, 0.85; 95% CI, 0.71 to 1.01; *P* = .069) and ZA (HR, 0.84; 95% CI, 0.70 to 1.00; *P* = .052), but no evidence of interaction between treatments in a further model (*P* = .942).

### CaP-Specific Survival

Of 584 deaths, 471 (81%) were a result of CaP; a higher proportion of deaths was attributed to CaP in patients with metastatic disease (381 of 459 deaths [83%] in 755 M1 patients, and 90 of 125 deaths [72%] in 490 patients with nonmetastatic disease). Adjusted competing risk regression for CaP-specific survival showed no evidence of advantage over SOC-only for SOC + Cel (sub-HR, 0.97; 95% CI, 0.77 to 1.23; *P* = .782) but an advantage for SOC + ZA + Cel (sub-HR, 0.74; 95% CI, 0.59 to 0.94; *P* = .014). For patients with metastatic disease, the sub-HR for SOC + Cel was 0.91 (95% CI, 0.71 to 1.18), and the sub-HR for SOC + ZA + Cel was 0.64 (95% CI, 0.49 to 0.83); for patients with nonmetastatic disease, the sub-HR for SOC + Cel was 1.44 (95% CI, 0.86 to 2.40), and the sub-HR for SOC + ZA + Cel was 1.31 (95% CI, 0.78 to 2.18).

Of deaths not related to CaP, 23 of 113 (20%) were classified as cardiovascular disease: nine of 52 (17%) SOC-only, three of 26 (12%) SOC + Cel, and 11 of 35 (31%) SOC + ZA + Cel.

### SSEs

A total of 207 of 622 SOC-only patients reported one or more SSEs. There was no evidence that the time to first SSE was improved with SOC + Cel (90 of 312 patients reported SSE: HR, 0.85; 95% CI, 0.66 to 1.08; *P* = .186) or with SOC + ZA + Cel (95 of 311 patients reported SSE: HR, 0.84; 95% CI, 0.66 to1.07; *P* = .162).

### AEs

In per protocol analyses of safety, one third reported worst AE ever as grade ≥ 3: 222 of 625 (36%) SOC-only, 98 of 296 (33%) SOC + Cel, and 95 of 293 (32%) SOC + ZA + Cel (Table 2). In 799 patients with AE assessment at approximately 1 year after random assignment, the proportions with grade ≥ 3 AE were 43 of 398 (11%) SOC-only, 16 of 200 (8%) SOC + Cel, and 13 of 196 (7%) SOC + ZA + Cel, mostly related to SOC with androgen deprivation therapy. Patterns and levels of AEs were similar in the ITT population. There were six reported cases of osteonecrosis of the jaw, all in the SOC + ZA + Cel group.

**Table 2.**
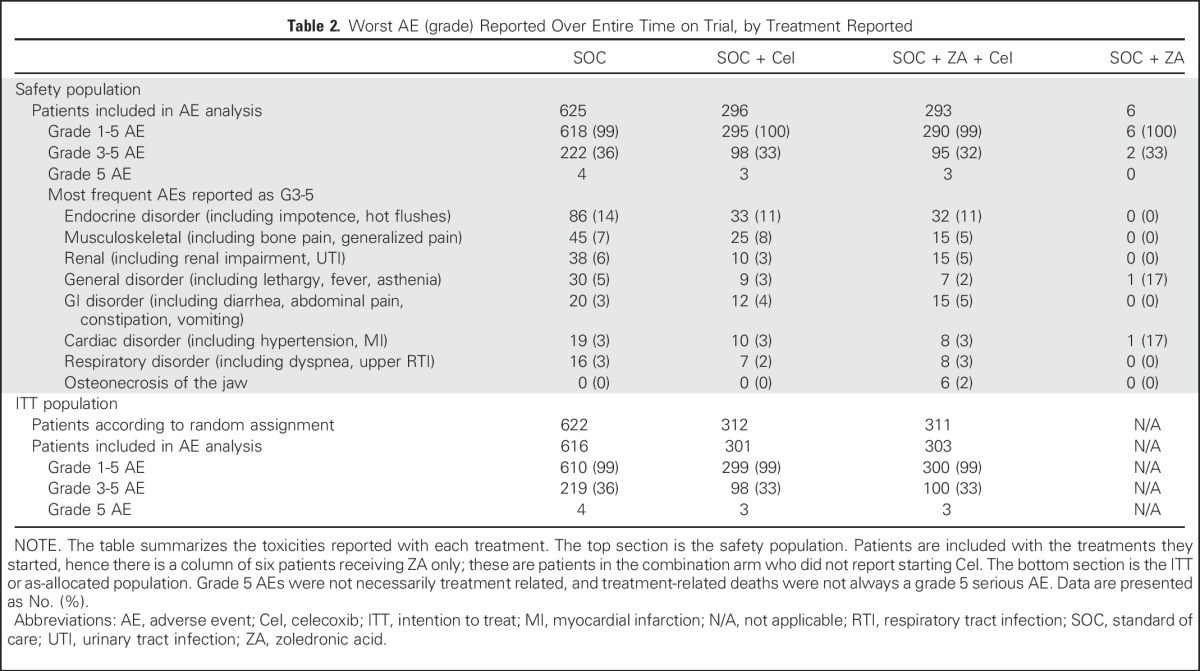
Worst AE (grade) Reported Over Entire Time on Trial, by Treatment Reported

### Second-Line Treatment

The Data Supplement lists time from FFS event to next treatment and time to any of the five life-extending therapies in castrate-refractory prostate cancer (available agents with proven survival gain). There was no evidence among arms of a difference in time to either any therapy or life-extending therapies (Data Supplement). There were no reports of patients allocated to SOC-only switching to Cel after progression; 82 and 22 patients in the SOC-only group and the SOC + Cel group, respectively, reported using ZA after progression.

## DISCUSSION

We report the findings of two randomized comparisons from STAMPEDE, a MAMS-platform trial, in 1,245 patients starting long-term first-line HT, with a median follow-up of > 5 years. These two Cel comparisons show no overall evidence that Cel, alone or combined with ZA, improved survival or FFS compared with SOC. However, a preplanned subgroup analysis suggested the possibility of benefit in terms of both survival and FFS for SOC + Cel + ZA over SOC alone in M1 patients at random assignment, although the test for interaction was not significant at a traditional 5% significance level.

Importantly, follow-up continued after accrual to Cel stopped. There is no bias in the reporting time of these comparisons; we report them, as planned, straight after survival results from the other arms that started contemporaneously. Data return rates are good, with most patients known to be alive having reported data in the past year.

Although recruitment to these comparisons was terminated early, there are still three quarters the number of control arm deaths that would have triggered this survival analysis had the comparisons passed all three interim analyses (n = approximately 300 of 400).

Results for the combination of Cel and ZA are intriguing and unexpected, given that we observed no evidence of improvement in OS or FFS with the addition of either Cel or ZA alone. Unfortunately, no further comparative data that would help directly in interpretation are expected. No other powered randomized controlled trial combining bisphosphonates and Cox-2 inhibitors are listed on trial registers; studies listed are nonrandomized or small or they terminated early. Our data fall short of definite evidence that this is a real effect; tests for interaction typically lack power, particularly in subgroups and where accrual is terminated early. Rather, they are hypothesis generating and provoke further research. Biologically, an effect by directly targeting tumor and/or host cells, particularly of stromal and immune lineage, is not implausible.^24-26^

The observed effect on survival of combined Cel and ZA in the metastatic setting is of similar magnitude to that reported for docetaxel in the STOPCaP (Systemic Treatment Options for Prostate Cancer) meta-analysis,^4^ although the observed effect on FFS is much less pronounced (Table 3 and Data Supplement). There are reports of anticancer activity with the nonsteroidal anti-inflammatory agent diclofenac,^24^ and STOPCaP suggests an overall benefit of bisphosphonates in metastatic disease, albeit driven by one trial of sodium clodronate.^4^

**Table 3.**
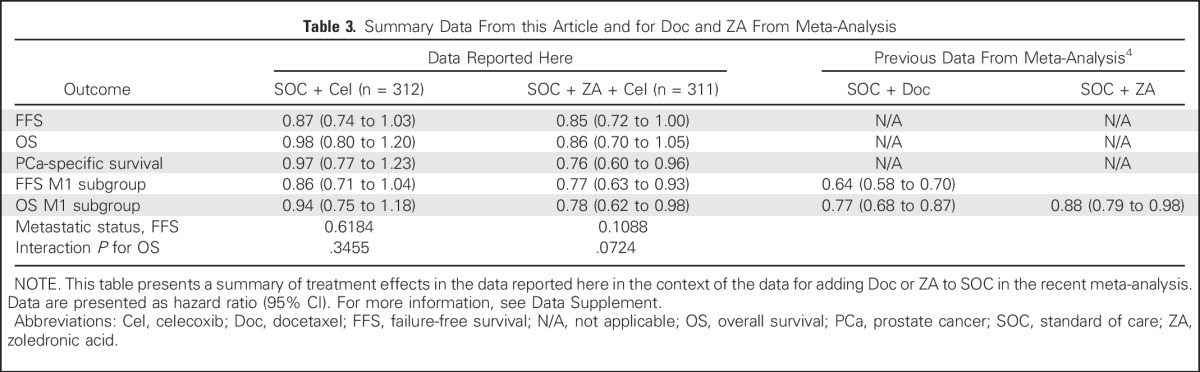
Summary Data From this Article and for Doc and ZA From Meta-Analysis

We defined survival as the definitive outcome measure, D, and FFS as the intermediate outcome measure, I. When this trial was first designed, some fundamental principles and assumptions were adopted, following the process for MAMS designs: (1) a treatment that does not improve I is unlikely to sufficiently improve D; (2) an improvement in I may not translate into an improvement in D; (3) therefore, a failure to improve I by a prespecified amount can be used as a triage for D, to allow early stopping of recruitment, but success in improving I does not remove the need to continue recruitment to reliably assess D; (4) I does not need to be a surrogate for D in the strictest sense, but only on the causal pathway; and (5) arms whose recruitment is stopped because of failure to improve I will continue to be followed up, and D will be reported soon after that for contemporaneously initiated arms.

When the trial launched in 2005, these assumptions were still thought to be reasonable with our choices for I and D. With the hindsight afforded by a decade of new knowledge and new data, we now realize that in CaP and in some other cancers, and with some interventions, there might be more discordance between FFS and survival in some circumstances.

FFS was the interim outcome measure for Cel and ZA. The vast majority of patients respond to HT with a fall in PSA values; we were specifically looking for an enhancement of the time to treatment failure by the addition of these agents, which legitimizes FFS as an outcome measure because response was not an appropriate outcome. The primary outcome measure is OS, which is not dependent directly on PSA values, although PSA values will have affected treatment decisions for clinicians along the way. The multistage approach in a MAMS trial does not require a perfect surrogate outcome measure, but one on the causal pathway, as FFS is for survival. Overall, there was minimal effect on FFS, and this translated into minimal effect on OS. In this regard, FFS was an acceptable choice. For the comparisons added later in STAMPEDE, we used the same definition of FFS treatment in both the abiraterone comparison and the enzalutamide plus abiraterone comparison in STAMPEDE, but allowed treatment to continue using all three types of progression (biochemical, clinical, and radiologic); for the new metformin comparison that opened in STAMPEDE in September 2016, we have chosen not to use a PSA-driven outcome measure as the intermediate outcome measure.^27,28^

Interpretation is further complicated by early cessation of Cel treatment when recruitment stopped at the second activity analysis (Data Supplement), which meant that fewer patients could receive the planned duration of their allocated treatment. Long-term estimation of the treatment effect on FFS of SOC + Cel versus SOC-only in the Cel comparison is consistent with our previous publication,^19^ when Cel failed to pass its second intermediate activity threshold.

At random assignment, RT was stated as part of the treatment plan for approximately one quarter of M0 patients across all arms. Around the time recruitment to these comparisons was stopped, data were emerging from NCIC-CTG-PR.3/MRC-PR07 and SPCG-7 that RT improved survival for men starting long-term HT for M0 CaP.^29,30^ Herein, the reported use of RT in the control group met expectations, but it was lower in both research arms. This preferential omission of RT from the Cel-containing arms complicates the findings, and a more favorable signal may have been observed had RT not been preferentially omitted for unrecorded reasons.

Approximately one half of the patients died, mostly as a result of CaP. There were several deaths with cardiovascular causes, but these occurred proportionally more in the combination arm.

One stratification factor at random assignment was the use of NSAIDs and/or aspirin, included only because of these Cel comparisons. Both comparisons had much improved FFS in patients receiving NSAIDs and/or aspirin at baseline, but this did not translate into evidence of a difference in survival. Baseline use of NSAIDs and/or aspirin likely related to underlying, pre-existing comorbidities, but this effect deserves further exploration.

Our data show no evidence of a survival advantage in adding Cel alone for all men starting long-term HT for the first time. We previously also showed no evidence of a survival advantage in adding ZA alone for the same patient group. Overall, the combination of Cel and ZA had no effect. Preplanned subgroup analyses may provide a hypothesis for future studies to investigate adding Cel in settings in which ZA is already part of the SOC.
